# Efficacy of pharmacological therapies for preventing post-dural puncture headaches in obstetric patients: a Bayesian network meta-analysis of randomized controlled trials

**DOI:** 10.1186/s12884-023-05531-7

**Published:** 2023-03-29

**Authors:** Ge Zhao, Guang Song, Jing Liu

**Affiliations:** 1 Department of Obstetrics, The First Hospital of China Medical University, No. 155 Nanjing North Street, Heping District, Shenyang, 110001 Liaoning Province China; 2 Department of Ultrasound, Shengjing Hospital of China Medical University, Shenyang, China

**Keywords:** Post-dural puncture headache, Pregnancy, Caesarean section, Randomized controlled trials, Meta-analysis

## Abstract

**Background:**

Post-dural puncture headache (PDPH) is a major complication of neuraxial anesthesia. PDPH usually occurs after Caesarean section in obstetric patients. The efficacy of prophylactic pharmacological therapies remains controversial.

**Methods:**

Seven pharmacological therapies (aminophylline (AMP), dexamethasone, gabapentin/pregabalin (GBP/PGB), hydrocortisone, magnesium, ondansetron (OND), and propofol (PPF)), were studied in this Bayesian network meta-analysis. The primary outcome was the cumulative incidence of PDPH within 7 days. Secondary outcomes included the incidence of PDPH at 24 and 48 h postoperatively, the severity of headache in PDPH patients (24, 48, and 72 h postoperatively), and postoperative nausea and vomiting (PONV).

**Results:**

Twenty-two randomized controlled trials with 4,921 pregnant women (2,723 parturients received prophylactic pharmacological therapies) were included. The analyses demonstrated that PPF, OND, and AMP were efficient in decreasing the cumulative incidence of PDPH during the follow-up period compared to the placebo group (OR = 0.19, 95% CI: 0.05 to 0.70; OR = 0.37, 95% CI: 0.16 to 0.87; OR = 0.40, 95% CI: 0.18 to 0.84, respectively). PPF and OND had the lower incidence of PONV compared to the placebo group (OR = 0.07, 95% CI: 0.01 to 0.30; and OR = 0.12, 95% CI: 0.02 to 0.63). No significant difference in other outcomes was found among different therapies.

**Conclusions:**

Based on available data, PPF, OND, and AMP may have better efficacy in decreasing the incidence of PDPH compared to the placebo group. No significant side effects were revealed. Better-designed studies are requested to verify these conclusions.

**Supplementary Information:**

The online version contains supplementary material available at 10.1186/s12884-023-05531-7.

## Introduction

Post-dural puncture headache (PDPH) is a serious complication of the neuraxial blockade that may arise after spinal anesthesia or epidural analgesia with an accidental dural puncture [1]. PDPH was first described in 1899 [2]. Previous studies demonstrated that PDPH had a wide range of incidences: 1.5% to 36% after spinal anesthesia [3–5]. A recent meta-analysis revealed that the incidence of PDPH was 23.47% in a total of 175,652 parturients who underwent Caesarean section with spinal anesthesia [6].

In spite of developments in needle design and puncture techniques, PDPH remains the most common complication of neuraxial blockade due to the popularity of neuraxial blockade in obstetric anesthesia [7]. Pregnancy, female sex, and young age are all established risk factors for PDPH, which are frequently observed in obstetric patients. PDPH symptoms often start within 48 to 72 h after the operation and resolve spontaneously within 1 week [8]. However, some PDPH can be delayed for months afterward. PDPH may develop as chronic postpartum headache [9–12], causing hearing loss [10, 13], backache [10–12], neckache [11], postpartum depression [11, 12], and decreased breastfeeding [12].

Several conservative treatments are recognized, including bed rest, hydration, and abdominal binder. However, bed rest may increase the risk of thromboembolic complications. Hydration and abdominal binder had insufficient evidence in the treatment of obstetric PDPH. Although there have been advances in the therapeutic epidural blood patch for PDPH treatment, it may cause chronic backache and a variety of neurological consequences [7, 14]. Given the foregoing, obstetric anesthetists are eager to explore effective prophylactic medicines.

Several pharmacological therapies for preventing PDPH have been developed in parturients, including aminophylline (AMP), dexamethasone (DXM), gabapentin/pregabalin (GBP/PGB), hydrocortisone (HCT), magnesium (Mg), ondansetron (OND), and propofol (PPF) [15–17]. However, the results were inconsistent. There have been few randomized controlled trials (RCTs) that directly compared these pharmacological therapies [18–21]. In this study, a network meta-analysis (NMA) was therefore conducted comparing these prophylactic pharmacological therapies among pregnant women during the perinatal period. This NMA aimed to provide a comprehensive overview of the efficacy of pharmacological therapies for preventing PDPH in obstetric patients in clinical practice.

## Methods

This systematic review was preregistered (https://www.crd.york.ac.uk/prospero/, ID: CRD42022346544).

### Search strategy

PubMed, MEDLINE, EMBASE, Scopus, ClinicalTrials.gov, Cochrane Library, and Google Scholar were searched (from database inception to July 25, 2022) to identify the available literature by two independent investigators (G.S. and G.Z.). The keywords were “post-dural puncture headache” and “pregnancy/Caesarean section” (Supplemental Table S1). In addition, citations of papers were examined to find other relevant literature.

### Eligibility criteria

Original studies were eligible if the following criteria were met: (i) was an RCT study; (ii) full text available in English; (iii) all participants were pregnant women; and (iv) assessed the efficacy of pharmacological therapies for preventing PDPH in parturients.

Original studies were ineligible for the following reasons: (i) observational studies, conference abstracts, or case reports; (ii) studies involving invasive therapies (i.e., prophylactic epidural blood patch or prophylactic intrathecal/subarachnoid morphine/fentanyl); (iii) lacked data to determine odds ratios (ORs) and 95% confidence interval (CI) of the efficacy of pharmacological therapies or mean difference and 95% CI of the severity of PDPH; or (iv) research on laboratory animals.

### Selection process and data extraction

Individual studies of NMA were first screened based on titles and abstracts. If a judgment could not be made based on titles and abstracts, we proceeded to read the full text. Both the screening process and data extraction were performed independently by two investigators (G.S. and G.Z.). Also, we used Cohen’s κ statistic for measuring inter-rater agreement. Senior investigators (J.L.) resolved discrepancies through discussions.

For each eligible MA, two independent investigators (G.Z. and J.L.) extracted data including: the first author, details of interventions, sample size, inclusion and exclusion criteria in each involved study, duration of follow-up, and outcomes. Data was obtained from the figures by using the GetData Graph Digitizer if they were not in the tables or full text [22].

### Quality assessment

The quality of the selected studies and risk of bias were assessed by the two independent reviewers (G.S. and G.Z.) using Cochrane Collaboration's tool [23]. Any disagreements were resolved by the senior reviewers (J.L.) or through consensus-based discussion. The quality evaluation charts were generated using the “robvis” package of the R software.

### Outcome definition

Cumulative incidence of PDPH within 7 days was the primary outcome. The incidence of PDPH at 24 and 48 h postoperatively, the severity of headache in PDPH patients (24, 48, and 72 h postoperatively), and side effects (such as postoperative nausea and vomiting (PONV)) were selected as secondary outcomes. Pain measures such as visual analogue scales represented the severity of PDPH. In this NMA, these pain measures were converted to an adjusted 0–10 point score ( “0” means no pain, and “10” means most serious pain) for further analysis [22].

### Statistical analysis

OR and 95% CI were used to report the incidence of PDPH and PONV. The severity of headache at different time points was reported as mean difference and 95% CI. The efficacy of pharmacological therapies for preventing PDPH in parturients was evaluated using an NMA. Random-effects and consistency models were used in the analysis (four chains, 50,000 iterations, and 20,000 per chain). Inconsistencies were reported if the Bayesian *P* values were greater than 0.05 after using the node-splitting method. Each therapy was given a rank based on the surface under the cumulative ranking curve (SUCRA) (worst = 0%; best = 100%) [24].

The GRADE method was applied to evaluate the overall quality of each outcome. Comparison-adjusted funnel plots were used to evaluate possible publication bias. The R software 3.6.3 (R Foundation, Vienna, Austria) with the “gemtc” package and Stata version 17.0 (StataCorp, College Station, TX, USA) was adopted.

## Results

### Study selection and study characteristics

**Fig. 1:**
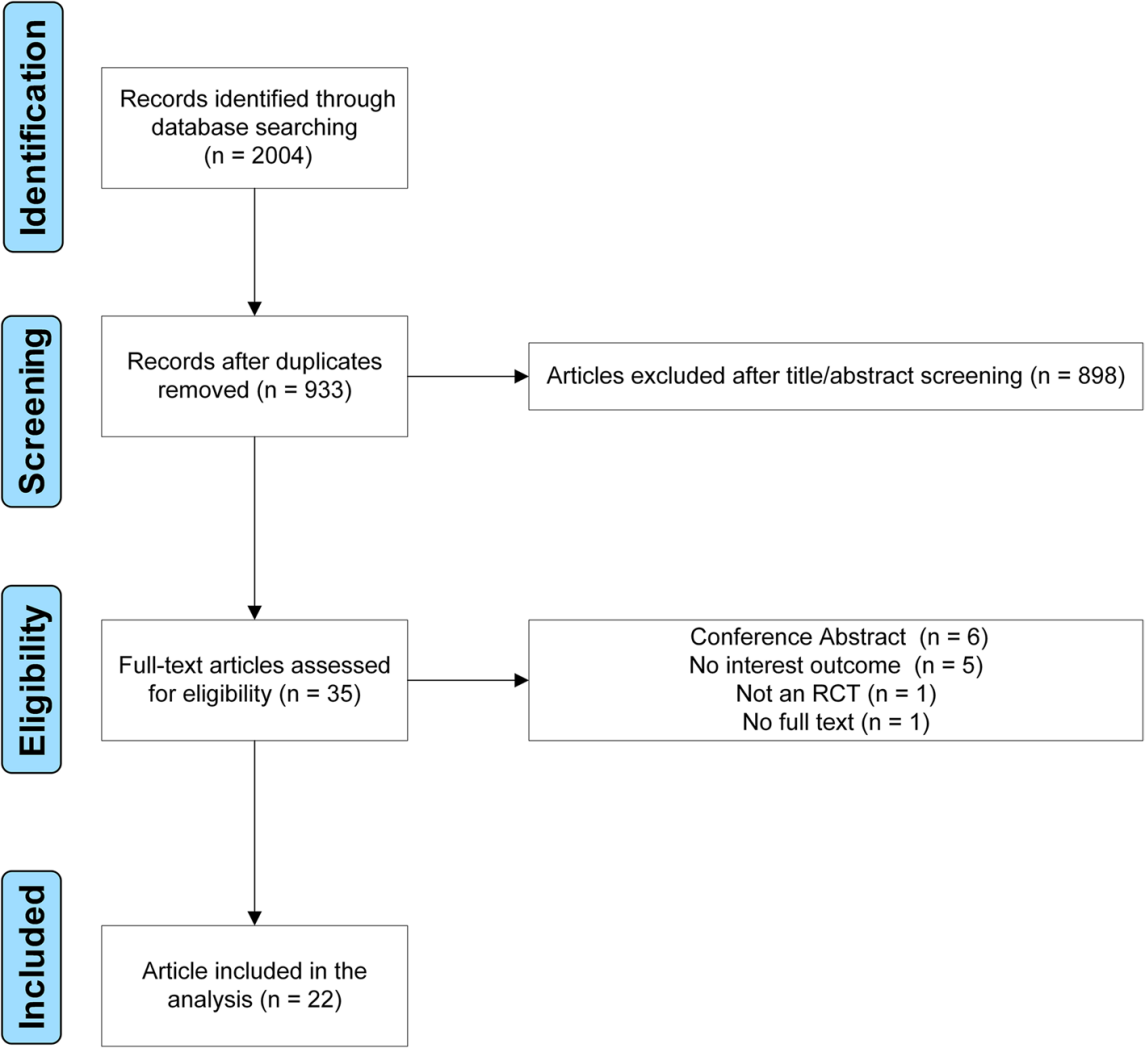
Flowchart of study selection. *RCT,* Randomized controlled trial

**Fig. 2:**
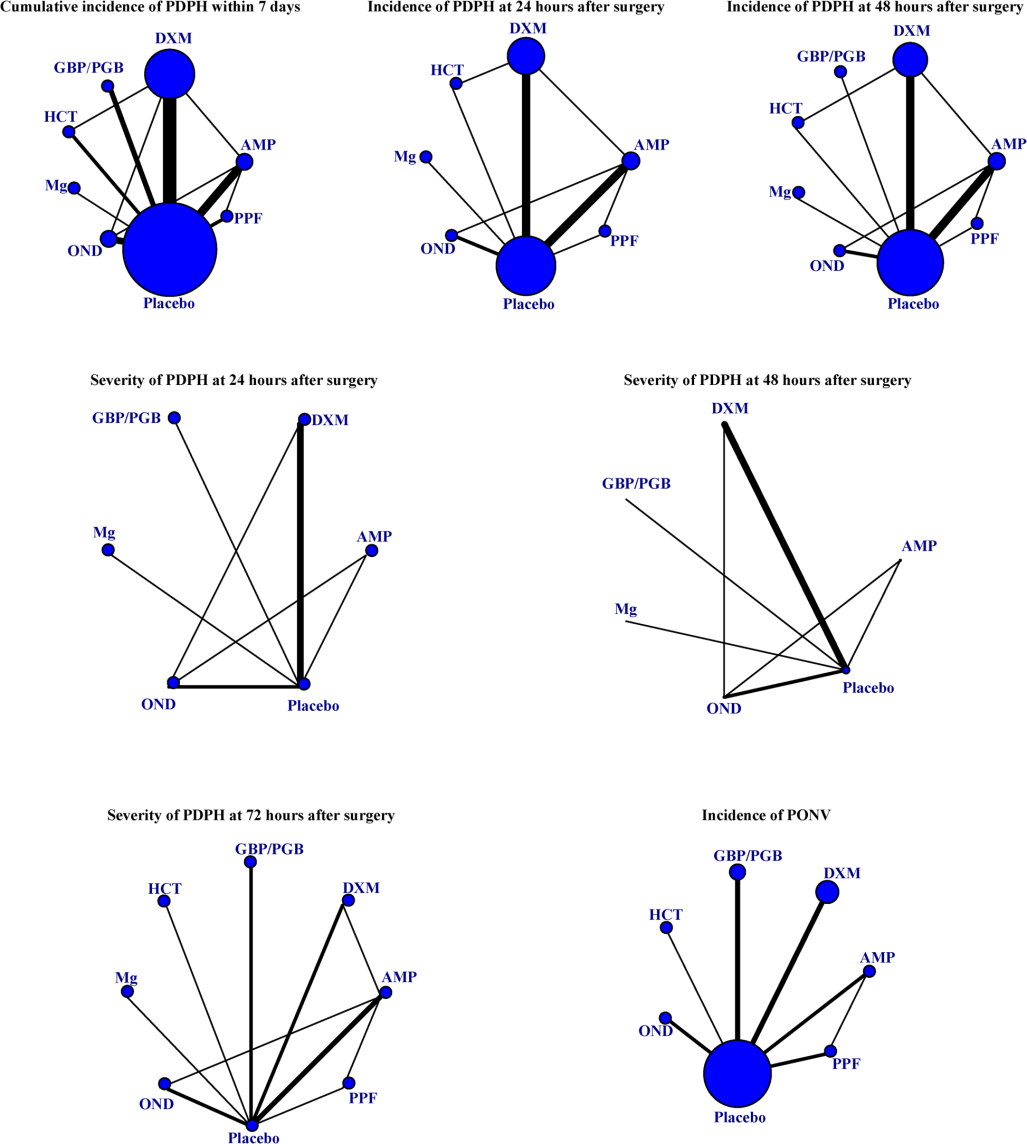
Network geometry. Circles represent the intervention as a node in the network. The size of the circle corresponds to the number of participants included in each comparison; lines represent direct comparisons using randomized controlled trials; and the thickness of the lines corresponds to the number of RCTs included in each comparison. *AMP,* Aminophylline*; DXM,* Dexamethasone*; GBP/PGB,* Gabapentin or pregabalin*; HCT,* Hydrocortisone*; Mg,* Magnesium*; OND,* Ondansetron*; PDPH,* Post-dural puncture headache*; PPF,* Propofol*; PONV,* Postoperative nausea and vomiting

Using an extensive search method, about 2,000 possibly relevant papers were gathered. Finally, 22 RCTs were included in our final analysis (Fig. 1) [18–21, 25–42]. The inter-rater agreement was very good for titles/abstracts and full-text screening (κ = 0.87 and 0.93, respectively). These trials were conducted between 2012 and 2022. There were 4,921 patients involved in this NMA, including 2,723 patients who received prophylactic pharmacological therapies (Table 1). Seven pharmacological therapies were assessed in these studies, including AMP, DXM, GBP/PGB, HCT, Mg, OND, and PPF (Fig. 2). All trials involved spinal anesthesia, and all patients underwent Caesarean section. Eighteen trials were two-arm studies; the other four trials were a three-arm design. The duration of follow-up ranged from 2 to 7 days. In addition, a summary of bias risk assessment was provided (Supplemental Figures S1 & S2).

**Table 1:** Characteristics of included studies

Author, year	Race	Anesthesia technique	Groups	Numbers	Interventions	Follow-up (days)	Outcomes
Hamzei, 2012 [25]	Asian	Spinal anesthesia	Dexamethasone	80	8 mg intravenous dexamethasone during surgery after delivery	7	①③④
			Placebo	80	No intervention		
Sadeghi, 2012 [26]	Asian	Spinal anesthesia	Aminophylline	60	1 mg/kg intravenous aminophylline during surgery after delivery	2	①②
			Placebo	60	No intervention		
Yousefshahi, 2012 [27]	Asian	Spinal anesthesia	Dexamethasone	182	8 mg (2 mL) intravenous dexamethasone during surgery after delivery	3	①②③④⑤⑦
			Placebo	178	2 mL normal saline		
Nofal, 2014 [28]	Caucasian	Spinal anesthesia	Gabapentin	42	Preoperative 600 mg of gabapentin capsules two hours before surgery	4	③④⑤⑥⑦
			Placebo	44	Starch capsules two hours before surgery		
Fattahi, 2015 [29]	Asian	Spinal anesthesia	Ondansetron	106	Intravenous ondansetron 0.15 mg/kg diluted in 5 mL normal saline during surgery	3	③⑥⑦
			Placebo	106	5 mL normal saline		
Ghanei, 2015 [30]	Asian	Spinal anesthesia	Aminophylline	100	2 mg/kg intravenous aminophylline during surgery after delivery	2	③⑦
			Placebo	100	Conventional therapy		
Mahmoud, 2015 [31]	Asian	Spinal anesthesia	Dexamethasone	289	4/8 mg (1/2 mL) of intravenous dexamethasone during surgery	7	③
			Placebo	155	2 mL normal saline		
Yang, 2015 [32]	Asian	Spinal anesthesia	Dexamethasone	307	8 mg (2 mL) intravenous dexamethasone during surgery after delivery	7	①②③④⑤⑥⑦
			Placebo	309	2 mL normal saline		
Golfam, 2016 [33]	Asian	Spinal anesthesia	Propofol	60	Intravenous propofol 30 µg/kg/min during surgery after delivery	7	③⑦
			Placebo	60	No intervention		
El–guoshy, 2018 [34]	Caucasian	Spinal anesthesia	Pregabalin	200	Oral pregabalin 150 mg before surgery	3	②③⑦
			Placebo	200	No intervention		
Pazoki, 2018 [35]	Asian	Spinal anesthesia	Ondansetron	127	4/8 mg of intravenous ondansetron before surgery	7	①②③⑦
			Placebo	64	Normal saline		
Shakhsemampour, 2018 [36]	Asian	Spinal anesthesia	Dexamethasone	52	2 mL intravenous dexamethasone before surgery	2	②⑤
			Placebo	52	2 mL normal saline		
Shokrpour, 2018 [18]	Asian	Spinal anesthesia	Ondansetron	40	8 mg intravenous ondansetron	2	③④⑤
			Dexamethasone	40	8 mg intravenous dexamethasone		
			Placebo	40	Distilled water		
Dehghanpisheh, 2019 [19]	Asian	Spinal anesthesia	Aminophylline	100	1 mg/kg intravenous aminophylline during surgery after delivery	3	①②③④⑤⑥
			Ondansetron	100	0.15 mg/kg intravenous ondansetron before surgery		
			Placebo	100	5 mL normal saline		
Yang, 2019 [37]	Asian	Spinal anesthesia	Aminophylline	59	250 mg intravenous aminophylline during surgery after delivery	7	①②③
			Placebo	58	Normal saline		
Anbarlouei, 2020 [38]	Asian	Spinal anesthesia	Dexamethasone	72	8 mg intravenous dexamethasone	7	①②③
			Hydrocortisone	72	200 mg intravenous hydrocortisone		
			Placebo	72	2 mL normal saline		
Ogunsiji, 2020 [39]	Black	Spinal anesthesia	Hydrocortisone	197	100 mg intravenous hydrocortisone diluted to 2 ml during surgery after delivery	5	③⑥⑦
			Placebo	194	2 mL normal saline		
Karami, 2021 [40]	Asian	Spinal anesthesia	Pregabalin	68	150 mg oral pregabalin at the night before spinal anesthesia	3	③⑥⑦
			Placebo	68	A placebo		
Refky, 2021 [20]	Caucasian	Spinal anesthesia	Propofol	52	30 μg/kg/min intravenous propofol in 50 mL saline during surgery after delivery	3	①②③⑥⑦
			Aminophylline	52	100 μg/kg/min intravenous aminophylline in 50 mL saline during surgery after delivery		
			Placebo	52	50 mL normal saline		
Nikooseresht, 2022 [41]	Asian	Spinal anesthesia	Magnesium	50	300 mg oral magnesium sachet 2 h before surgery	5	①②③④⑤⑥
			Placebo	50	Starch powder 2 h before surgery		
Okpala, 2020 [42]	Black	Spinal anesthesia	Dexamethasone	96	8 mg (2 mL) intravenous dexamethasone during surgery after delivery	4	③⑦
			Placebo	96	2 mL normal saline		
Razavizadeh, 2022 [21]	Asian	Spinal anesthesia	Aminophylline	60	1.5 mg/kg intravenous aminophylline (5 mL) during surgery after delivery	7	①②③⑥
			Dexamethasone	60	0.1 mg/kg intravenous dexamethasone (5 mL) during surgery after delivery		
			Placebo	60	5 mL normal saline		

*NR* Not reported

①Incidence of post-dural puncture headache at 24 h after surgery

②Incidence of post-dural puncture headache at 48 h after surgery

③Cumulative incidence of post-dural puncture headache within 7 days

④Severity of post-dural puncture headache at 24 h after surgery

⑤Severity of post-dural puncture headache at 48 h after surgery

⑥Severity of post-dural puncture headache at 72 h after surgery

⑦Incidence of postoperative nausea and vomiting

### Cumulative incidence of PDPH within 7 days

Twenty trials reported a difference in cumulative incidence of PDPH within 7 days among pharmacological therapies and placebo groups [18–21, 25, 27–35, 37–42]. Of these 4,697 pregnant women, 2,611 received pharmacological therapies, and 2,086 received the placebo treatment. The incidence of PDPH was 15.2% (398/2611) in the pharmacological groups and 22.6% (471/2,086) in the placebo group.

**Fig. 3:**
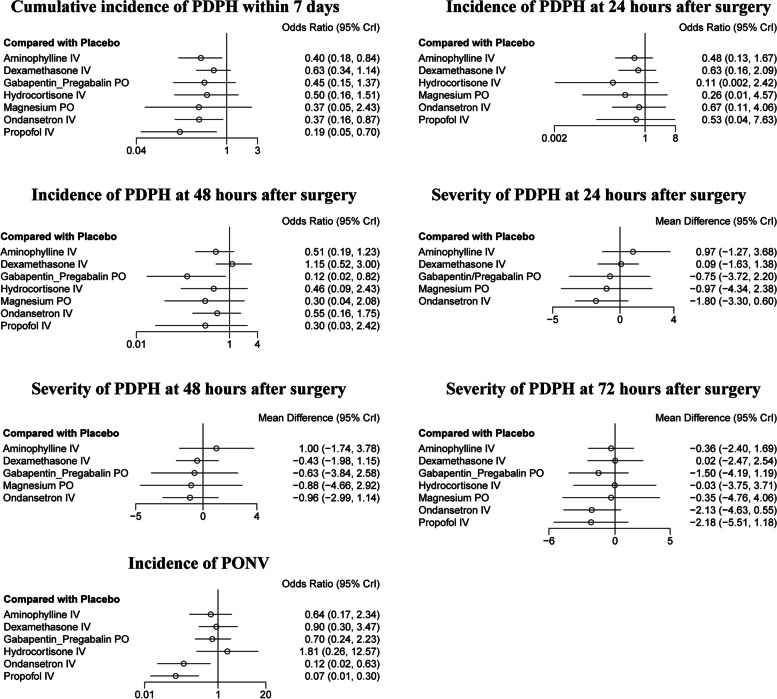
Forest plots of network meta-analysis of all outcomes. *AMP,* Aminophylline*; DXM,* Dexamethasone*; GBP/PGB,* Gabapentin or Pregabalin*; HCT,* Hydrocortisone*; IV,* Ontravenous*; Mg,* Magnesium*; OND,* Ondansetron*; PDPH,* Post-dural puncture headache*; PPF,* Propofol*; PO,* Oral*; PONV,* Postoperative nausea and vomiting

**Fig. 4:**
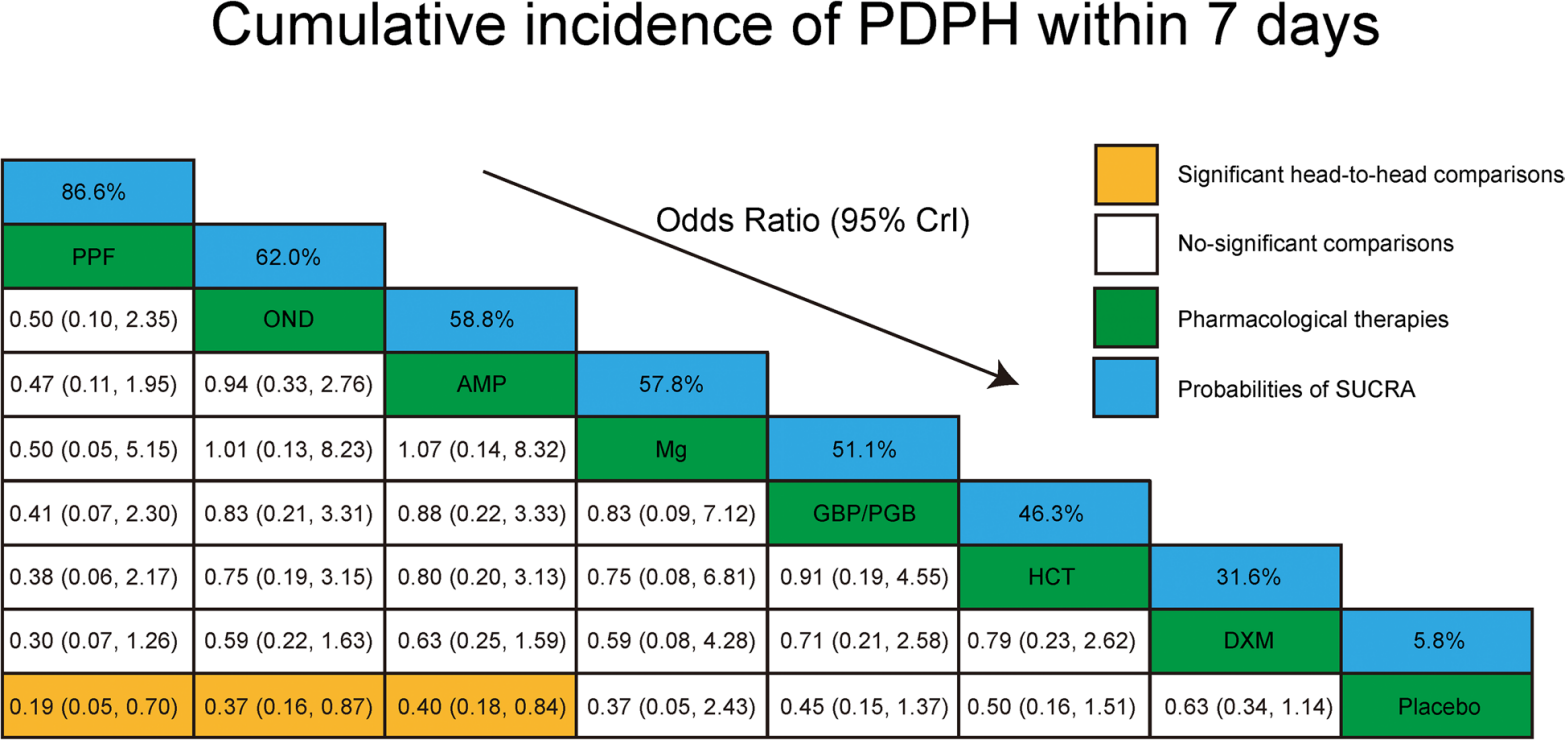
Head-to-head comparisons of incidence of post-dural puncture headache during the follow-up period. Highest probability of being the most efficient therapies (with high SUCRA values) and lowest probability of being the most efficient therapies (with low SUCRA values). *AMP,* Aminophylline*; DXM,* Dexamethasone*; GBP/PGB,* Gabapentin or Pregabalin*; HCT,* Hydrocortisone*; Mg,* Magnesium*; OND,* Ondansetron*; PDPH,* Post-dural puncture headache*; PPF,* Propofol*; SUCRA,* Surface under the cumulative ranking area curves

As shown in Figs. 3 and 4, the results of the NMA demonstrated that PPF, OND, and AMP were efficient in decreasing the incidence of PDPH compared to the placebo group (OR = 0.19, 95% CI: 0.05 to 0.70; OR = 0.37, 95% CI: 0.16 to 0.87; OR = 0.40, 95% CI: 0.18 to 0.84, respectively).

### Comparison of secondary outcomes

GBP/PGB decreased the incidence of PDPH at 48 h after surgery compared to the placebo group (OR = 0.12, 95% CI: 0.02 to 0.82, Fig. 3, Supplemental Table S3). No difference in the incidence of PDPH at 24 h after surgery was found among different therapies (Supplemental Table S4). No difference in the severity of PDPH (pain scores) was found at any time point among different therapies (Supplemental Tables S5-S7).

Twelve studies reported side effects during the perioperative period [20, 27–30, 32–35, 39, 40, 42]. All twelve studies reported PONV [20, 27–30, 32–35, 39, 40, 42]. Meanwhile, the results demonstrated that PPF and OND had lower incidence of PONV compared to the placebo group (OR = 0.07, 95% CI: 0.01 to 0.30; and OR = 0.12, 95% CI: 0.02 to 0.63, Fig. 3, Supplemental Table S8). Sedation [28], diplopia [33], and tinnitus [33] were also mentioned in individual studies, but were not involved in the qualitative synthesis. Dexamethasone did not show any superiority to other pharmacological therapies or to placebo in any secondary outcomes.

### Inconsistency, ranking, and publication bias

No inconsistency was found in any of the outcomes (all *P* > 0.05) (Supplemental Figures S3-S9). The ranking of each pharmacological therapy was performed and presented (Table 2, Fig. 4). There was no publication bias identified (Supplemental Table S9 and Supplemental Figure S10).

**Table 2:** GRADE quality score assessment for the outcomes

Outcome	Study number	Participants number	Rank of SUCRA	GRADE Quality score
Cumulative incidence of post-dural puncture headache within 7 days	20	4697	PPF (86.6%) > OND (62.0%) > AMP (58.8%) > Mg (57.8%) > GBP/PGB (51.1%) > HCT (46.3%) > DXM (31.6%) > Placebo (5.8%)	Moderate ^a^
Incidence of post-dural puncture headache at 24 h after surgery	11	2516	HCT (81.1%) > Mg (65.8%) > AMP (53.6%) > PPF (46.7%) > DXM (42.7%) > OND (40.1%) > Placebo (20.0%)	Moderate ^a^
Incidence of post-dural puncture headache at 48 h after surgery	12	2860	GBP/PGB (88.2%) > Mg (66.1%) > PPF (64.8%) > HCT (53.3%) > AMP (50.9%) > OND (47.0%) > Placebo (17.2%) > DXM (12.4%)	Moderate ^a^
Severity of post-dural puncture headache at 24 h after surgery	7	182	OND (85.1%) > Mg (64.6%) > GBP/PGB (60.9%) > Placebo (40.7%) > DXM (38.1%) > AMP (10.6%)	Moderate ^a^
Severity of post-dural puncture headache at 48 h after surgery	7	161	OND (71.5%) > Mg (62.1%) > GBP/PGB (58.1%) > DXM (54.8%) > Placebo (37.3%) > AMP (16.1%)	Moderate ^a^
Severity of post-dural puncture headache at 72 h after surgery	9	294	OND (78.6%) > PPF (77.5%) > GBP/PGB (66.3%) > Mg (42.2%) > AMP (40.0%) > HCT (35.2%) > DXM (31.3%) > Placebo (28.9%)	Moderate ^a^
Postoperative nausea and vomiting	12	2781	PPF (94.3%) > OND (86.1%) > AMP (49.1%) > GBP/PGB (45.8%) > DXM (34.1%) > Placebo (27.3%) > HCT (13.4%)	Moderate ^a^

*AMP* Aminophylline, *DXM* Dexamethasone, *GBP/PGB* Gabapentin or pregabalin, *HCT* Hydrocortisone, *Mg* Magnesium, *OND* Ondansetron, *PPF* Propofol

^a^ Rated down for serious imprecision

## Discussion

This is the first NMA regarding the efficacy of pharmacological therapies for preventing PDPH in obstetric patients who underwent Caesarean sections. A large amount of evidence was pooled to make it possible to indirectly compare the efficacy of these seven medicines. Analysis demonstrated that PPF, OND, and AMP could decrease the incidence of PDPH. No obvious side effects were revealed in these analyses or in the involved studies. These are encouraging findings.

The pathophysiology of PDPH is uncertain. There are three hypothesized mechanisms: compensatory meningeal venodilation and blood volume increase induced by cerebrospinal fluid (CSF) leak hypotension, leading to acute intracranial dilatation and headaches [43, 44]; CSF leak hypotension causing brain tissue to sag and nerves to stretch, resulting in headaches [45, 46]; and spinal puncture changing craniospinal elasticity, resulting in increasing caudal compliance and acute intracranial dilatation [47].

PPF is a γ-aminobutyric acid receptor and ultra-short-acting anesthetic [48]. PPF has favorable pharmacokinetic and pharmacodynamic characteristics and has become one of the most commonly used intravenous anesthetics [49]. Previous studies have demonstrated the efficacy of PPF in treating migraine [48, 50–52]. Soleimanpour et al. performed an RCT and proved that intravenous PPF was a more effective and safer treatment than DXM for patients presenting with migraine headaches [52]. Later, Golfam [33], Refky [20], and their colleagues attempted to use PPF for preventing PDPH in obstetric patients. The mechanism of PPF in PDPH prevention still needs further study. Meanwhile, PPF decreased the risk of PONV, which was consistent with previous studies [53, 54].

OND, a specific 5-HT 3 receptor antagonist, is frequently used for the prevention and management of PONV [29]. Four studies focused on the prophylactic effect on PDPH [18, 19, 29, 35]. The following mechanism of OND in PDPH prevention has been proposed: by inhibiting 5-HT3 receptors, OND reduced acute intracranial dilatation and maintained mean arterial pressure, which prevented compensatory intracranial vasodilation through autoregulation of cerebral circulation [29]. This effect might reduce the incidence of PDPH in parturients. A very low-probability complication requires doctors to be vigilant: OND or palonosetron may induce migraine headaches among those parturients who have experienced migraines, according to findings from two case reports [55–57].

As a theophylline active metabolite, AMP is a well-known methylxanthine medication. Previous studies showed that theophylline [58] and caffeine [59] might prevent PDPH by adenosine antagonization and vasoconstriction. Therefore, some doctors have tried to explore its efficacy in preventing PDPH among women experiencing Caesarean sections [19–21, 26, 30, 37]. However, they obtained conflicting results. Three trials found positive results [26, 30, 37], and the other three had negative results [19–21]. A meta-analysis, published in 2021, revealed that AMP could not prevent PDPH, but decreased pain scores in individuals who underwent different surgeries under spinal anesthesia and developed PDPH [16]. The findings in this study are contrary to this meta-analysis, which has suggested that further large-scale studies are warranted to confirm our result.

Only a few trials have focused on the efficacy of GBP [28], PGB [34, 40], and Mg [41]. All these therapies need more raw data to draw solid conclusions. Finally, the results of this study revealed that DXM and HCT, as the two most common glucocorticoids, were unable to reduce the incidence and severity of PDPH in parturients, which was consistent with the recent meta-analysis [17].

### Strengths and limitations

Considering all the above-described available options, the main objective of this study was to determine the best prophylactic pharmacological therapies for preventing PDPH after Caesarean section. The current meta-analysis had some limitations. First, there were only a few well-designed RCTs in this NMA. For example, some studies failed to reveal the details of allocation concealment. Second, needle type/size, the direction of bevel of the needle, angle of approach, and number of attempts may increase heterogeneity and affect the credibility of the conclusions. Third, the characteristics of parturients, such as maternal age, body mass index, and history of headache, were all underlying confounders. Fourth, variations in the dose of pharmacological therapies, type of placebo, and the duration of follow-up may bias the results. Fifth, the incidences of PDPH in the placebo group in the involved studies varied over a wide range. For a disease with a low incidence, it was difficult to find the difference in efficacy between two drugs in a small sample. Therefore, large sample-sized RCTs are needed in future to confirm our findings.

## Conclusions

Based on available data, PPF, OND, and AMP may have better efficacy than other proposed treatments in decreasing the incidence of PDPH. No obvious side effects were revealed in the analyses or the involved studies. Better-designed RCTs are needed to validate the conclusions.

## Supplementary Information

**Additional file 1.** PRISMA Network Meta-analysis Checklist.

**Additional file 2:** **Table S1.** Strategy of this meta-analysis.** Table S2.** Inclusion and exclusion criteria in each involved study.** Table S3.** Head-to-head comparisons of incidence of post-dural puncture headache at 48 hours after surgery.** Table S4.** Head-to-head comparisons of incidence of post-dural puncture headache at 24 hours after surgery.** Table S5.** Head-to-head comparisons of severity of post-dural puncture headache at 24 hours after surgery.** Table S6.** Head-to-head comparisons of severity of post-dural puncture headache at 48 hours after surgery.** Table S7.** Head-to-head comparisons of severity of post-dural puncture headache at 72 hours after surgery.** Table S8.** Head-to-head comparisons of incidence of postoperative nausea and vomiting.** Table S9.** Assessment of publication bias for network meta-analysis.** Figure S1.** Risk of bias summary.** Figure S2.** Risk of bias graph.** Figure S3.** Inconsistency test of cumulative incidence of post-dural puncture headache within 7 days.** Figure S4.** Inconsistency test of incidence of post-dural puncture headache at 24 hours after surgery.** Figure S5.** Inconsistency test of incidence of post-dural puncture headache at 48 hours after surgery.** Figure S6.** Inconsistency test of severity of post-dural puncture headache at 24 hours after surgery.** Figure S7.** Inconsistency test of severity of post-dural puncture headache at 48 hours after surgery.** Figure S8.** Inconsistency test of severity of post-dural puncture headache at 72 hours after surgery.** Figure S9.** Inconsistency test of postoperative nausea and vomiting.** Figure S10.** Funnel plot of the outcomes.

## Data Availability

The datasets used and/or analyzed during the current study are available from the corresponding author on reasonable request.

## Abbreviations

AMP: Aminophylline
CI: Confidence interval
DXM: Dexamethasone
GBP/PGB: Gabapentin/pregabalin
HCT: Hydrocortisone
MD: Mean difference
Mg: Magnesium
NMA: Network meta-analysis
OND: Ondansetron
OR: Odds ratio
PDPH: Post-dural puncture headache
PONV: Postoperative nausea and vomiting
PPF: Propofol
RCT: Randomized controlled trial
SUCRA: Surface under the cumulative ranking curve
