# Supplementary material for: Efficacy of pharmacological therapies for preventing post-dural puncture headaches in obstetric patients: a Bayesian network meta-analysis of randomized controlled trials
Source: BMC Pregnancy Childbirth. 2023 Mar 29;23:215. doi: 10.1186/s12884-023-05531-7 (PMC10053677; doi:10.1186/s12884-023-05531-7)

Efficacy of Pharmacological Therapies for Preventing Post-Dural Puncture Headache in Obstetric Patients: A Bayesian network meta-analysis of randomized controlled trials

Content

Table S1. Strategy of this meta-analysis 2

Table S2. Inclusion and exclusion criteria in each involved study 3

Table S3. Head-to-head comparisons of incidence of post-dural puncture headache at 48 hours after surgery 4

Table S4. Head-to-head comparisons of incidence of post-dural puncture headache at 24 hours after surgery 4

Table S5. Head-to-head comparisons of severity of post-dural puncture headache at 24 hours after surgery 4

Table S6. Head-to-head comparisons of severity of post-dural puncture headache at 48 hours after surgery 5

Table S7. Head-to-head comparisons of severity of post-dural puncture headache at 72 hours after surgery 5

Table S8. Head-to-head comparisons of incidence of postoperative nausea and vomiting 5

Table S9. Assessment of publication bias for network meta-analysis 6

Figure S1. Risk of bias summary 7

Figure S2. Risk of bias graph 8

Figure S3. Inconsistency test of cumulative incidence of post-dural puncture headache within 7 days 9

Figure S4. Inconsistency test of incidence of post-dural puncture headache at 24 hours after surgery 9

Figure S5. Inconsistency test of incidence of post-dural puncture headache at 48 hours after surgery 9

Figure S6. Inconsistency test of severity of post-dural puncture headache at 24 hours after surgery 10

Figure S7. Inconsistency test of severity of post-dural puncture headache at 48 hours after surgery 10

Figure S8. Inconsistency test of severity of post-dural puncture headache at 72 hours after surgery 10

Figure S9. Inconsistency test of postoperative nausea and vomiting 11

Figure S10. Funnel plot of the outcomes 11

Table S1. Strategy of this meta-analysis

	Strategy
**PubMed**	#1: randomized controlled trial[Publication Type] OR controlled clinical trial[Publication Type] OR "Randomized Controlled Trials as Topic"[Mesh] OR randomized[Title/Abstract] OR random[Title/Abstract] OR randomly[Title/Abstract] OR controlled[Title/Abstract] OR trial[Title/Abstract] OR placebo[Title/Abstract] OR groups[Title/Abstract]<br>#2: "Post-Dural Puncture Headache*"[Mesh] OR post-dural puncture headache*[Title/Abstract] OR postdural puncture headache*[Title/Abstract] OR post dural puncture headache*[Title/Abstract] OR PDPH[Title/Abstract] OR (lumbar puncture headache*[Title/Abstract] OR Post-lumbar puncture headache*[Title/Abstract] OR post-LP headache*[Title/Abstract]) OR ("Spinal Puncture"[Mesh] OR post-spinal puncture headache*[Title/Abstract] OR spinal tap headache*[Title/Abstract])<br>#3: "Pregnancy"[Mesh] OR pregnan*[Title/Abstract] OR gestation*[Title/Abstract] OR "Cesarean Section"[Mesh] OR cesarean[Title/Abstract] OR caesarean[Title/Abstract] OR parturient*[Title/Abstract] OR labour*[Title/Abstract] OR labor*[Title/Abstract] OR obstetric*[Title/Abstract]<br>#4: #1 AND #2 AND #3 AND (english[Filter])
**MEDLINE**	#1: (Randomized Controlled Trials as Topic.sh. OR randomized controlled trial.pt OR controlled clinical trial.pt OR randomized.mp. OR placebo.mp. OR random.mp. OR randomly.mp. OR controlled.mp. OR trial.mp. OR groups.mp. OR Clinical Trials as Topic/)<br>#2: Post-Dural Puncture Headache.sh. OR post-dural puncture headache*.mp. OR postdural puncture headache*.mp. OR post dural puncture headache*.mp. OR PDPH.mp. OR (lumbar puncture headache*.mp. OR Post-lumbar puncture headache*.mp. OR post-LP headache*.mp.) OR (Spinal Puncture.sh. OR post-spinal puncture headache*.mp. OR spinal tap headache*.mp.)<br>#3: Pregnancy.sh. OR Cesarean Section.sh. OR (pregnan* OR gestation* OR cesarean OR caesarean OR parturient* OR labour* OR labor* OR obstetric*)<br>#4: #1 AND #2 AND #3<br>#5: limit 4 to (english language)
**Embase**	#1：'randomized controlled trial':it OR 'controlled clinical trial':it OR 'Randomized Controlled Trials as Topic'/exp OR 'randomized':ab,ti OR 'random':ab,ti OR 'randomly':ab,ti OR 'controlled':ab,ti OR 'trial':ab,ti OR 'placebo':ab,ti OR 'groups':ab,ti<br>#2：'Post-Dural Puncture Headache*'/exp OR 'post-dural puncture headache*':ab,ti OR 'postdural puncture headache*':ab,ti OR 'post dural puncture headache*':ab,ti OR 'PDPH':ab,ti OR ('lumbar puncture headache*':ab,ti OR 'Post-lumbar puncture headache*':ab,ti OR 'post-LP headache*':ab,ti) OR ('Spinal Puncture'/exp OR 'post-spinal puncture headache*':ab,ti OR 'spinal tap headache*':ab,ti)<br>#3：'Pregnancy'/exp OR 'pregnan*':ab,ti OR 'gestation*':ab,ti OR 'Cesarean Section'/exp OR 'cesarean':ab,ti OR 'caesarean':ab,ti OR 'parturient*':ab,ti OR 'labour*':ab,ti OR 'labor*':ab,ti OR 'obstetric*':ab,ti<br>#4: #1 AND #2 AND #3
**Scopus**	TITLE-ABS-KEY (“randomized” OR “random” OR “randomly” OR “controlled” OR “trial” OR “placebo” OR “groups”) AND TITLE-ABS-KEY (“Post-Dural Puncture Headache*” OR “postdural puncture headache*” OR “post dural puncture headache*” OR “PDPH” OR “lumbar puncture headache*” OR “Post-lumbar puncture headache*” OR “post-LP headache*” OR “Spinal Puncture” OR “post-spinal puncture headache*” OR “spinal tap headache*”) AND TITLE-ABS-KEY (“pregnan*” OR “gestation*” OR “cesarean” OR “caesarean” OR “parturient*” OR “labour*” OR “labor*” OR “obstetric*”) AND LANGUAGE(english) AND DOCTYPE(ar) NOT INDEX(medline)
**ClinicalTrials.gov**	Post-Dural Puncture Headache| Interventional Studies | Studies with Female Participants
**Cochrane library**	#1 randomized controlled trial 1139156<br>#2 controlled clinical trial 1080780<br>#3 Randomized Controlled Trials as Topic 61838<br>#4 randomized 1261658<br>#5 random 83109<br>#6 randomly 285931<br>#7 controlled 1895152<br>#8 trial 1400286<br>#9 placebo 352815<br>#10 groups 540350<br>#11 #1 OR #2 OR #3 OR #4 OR #5 OR #6 OR #7 OR #8 OR #9 OR #10 1897549<br>#12 Post-Dural Puncture Headache* 406<br>#13 postdural puncture headache* 564<br>#14 post dural puncture headache* 437<br>#15 PDPH 297<br>#16 lumbar puncture headache* 335<br>#17 Post-lumbar puncture headache* 56<br>#18 post-LP headache* 6<br>#19 Spinal Puncture 1476<br>#20 post-spinal puncture headache* 37<br>#21 spinal tap headache* 26<br>#22 #12 OR #13 OR #14 OR #15 OR #16 OR #17 OR #18 OR #19 OR #20 OR #21 1756<br>#23 pregnan* 76610<br>#24 gestation* 29409<br>#25 caesarean 15412<br>#26 cesarean 15411<br>#27 parturient* 2653<br>#28 labour* 6709<br>#29 labor* 93798<br>#30 obstetric* 43777<br>#31 #23 OR #24 OR #25 OR #26 OR #27 OR #28 OR #29 OR #30 188481<br>#32 #11 AND #22 AND #31 591<br>#33 #32 NOT (EMBASE OR Pubmed) 249
**Google Scholar**	“randomized controlled trial” AND “Post-Dural Puncture Headache” AND “Cesarean Section”

Table S2. Inclusion and exclusion criteria in each involved study

Author, year	Groups	Age (years)	Inclusion criteria	Exclusion criteria
**Hamzei, 2012**	Dexamethasone<br>Placebo	20–40	Having undergone cesarean section under spinal anesthesia, having no history of chronic headaches or migraines, or no known emotional or mental illness; and falling within the American Society of Anesthesiologists 14 physical condition class ofemergency-1and condition-1.	For all patients, only one anesthesiologist performed the spinal anesthesia with only one try, and if it was necessary to more than one time that sample were excluded from the study. Furthermore, participants who underwent 2 anesthetics during the period were excluded.
**Sadeghi, 2012**	Aminophylline<br>Placebo	26.1±4.5<br>26.4±5.3	Undergoing elective cesarean section; performing the spinal anesthesia	The patients who have headache, psychiatric problems, back pain, preeclampsia, coagulation disorders, convulsion background, spinal anesthesia history and those who used any kinds of opiates were excluded from the study.
**Yousefshahi, 2012**	Dexamethasone<br>Placebo	28.5<br>28.9	Undergo cesarean section under spinal anesthesia	Patients with ASA class higher than II, who refused to attend the study, were sensitive to local anesthetics,<br>received anticoagulant therapy, or had experienced preeclampsia or skin infection at the site of needle insertion were exclude.
**Nofal, 2014**	Gabapentin<br>Placebo	32.1±4.8<br>30.7±5.2	Anesthesiologist I and II primi or multigravida women, pregnant in a single full-term fetus, and who were planned for elective cesarean section under spinal anesthesia.	We excluded women with history of chronic headache or any type of chronic pain and those on regular analgesics or antiepileptic medications. Women with contraindications to spinal anesthesia, to gabapentin, or to any other medication in this study were also excluded. In addition, in case of any abnormalities in the fetus, the patient was excluded.
**Fattahi, 2015**	Ondansetron<br>Placebo	24.1±2.0<br>25.1±2.1	Elective cesarean section under spinal anesthesia.	Having a history of cardiovascular disorders, migraine headache, taking selective serotonin reuptake inhibitors, hypersensitivity to ondansetron and local anesthetic drugs, and having contraindication for spinal anesthesia.
**Ghanei, 2015**	Aminophylline<br>Placebo	20–40	ASA class I or II who were planned for elective caesarean section under spinal anesthesia	Having history of history of migraine, cluster headache, contraindication to caesarean section surgery and spinal anesthesia, hypo or hyperthyroidism, sever preeclampsia, a height <152 cm, preoperative body temperature>38℃, Reynaud's syndrome, and allergy to the study medications (Aminophylline).
**Mahmoud, 2015**	Dexamethasone<br>Placebo	27.2±4.9<br>27.5±4.9	Full term pregnant female indicated for elective section under spinal anesthesia with no history of medical importance.	Urgent cases, emergency cases, adverse medical history, anticoagulant treatment, associated pregnancy complication, failed spinal, surgical complication or refusal was excluded.
**Yang, 2015**	Dexamethasone<br>Placebo	29.9±3.9<br>30.0±4.4	Undergoing cesarean section under spinal anesthesia; and American Society of Anesthesiologists (ASA) grade of I–II.	The subjects were excluded if they had a history of dexamethasone intolerance or past hypersensitivity reaction to it, a past history of chronic headaches or recent onset acute headaches and/or a history of known emotional or mental illness. Patients that had experienced pre-eclampsia or skin infections at the site of needle insertion were also excluded.
**Golfam, 2016**	Propofol<br>Placebo	30.5±6.6<br>31.7±7.7	ASA CLASS I and II, age range of 18-45, BMI of 23-27, and height of 160-170.	Comprised of history of headache, history of using analgesics, drug and tobacco addiction, presence of cardiac, respiratory and mental diseases, diabetes, other diseases requiring therapeutical measures (e.g. preeclampsia), patients with more than one attempt for needle insertion into dura, and patients with more than 1 Lit (20%) bleeding during surgery.
**El–guoshy, 2018**	Pregabalin<br>Placebo	NR	ASA class I or II patients. Patients undergoing<br>elective cesarean section.	ASA III, IV and V class patients. Patients who had history of convulsion. Patients with known allergy to local anesthetic or to the study drug. Patients who had any contraindications to regional anesthesia (e.g., patient refusal, local infection, coagulation abnormality and tight mitral stenosis). Patients with chronic headache. Patients undergoing urgent cesarean section.
**Pazoki, 2018**	Ondansetron<br>Placebo	29.6±5.2<br>29.4±4.9	ASA I-II, 15 age of 20-35 years, patient consent, no history of cardiovascular disease (cardiac arrhythmia, ischemia and heart failure), no SSRI prescriptions, no opioid prescriptions, lack of preeclampsia and eclampsia, and no history of migraine and diabetes.	Had any history of cardiovascular disease, migraine headache, use of selective serotonin reuptake inhibitors (SSRIs), sensitivity to ondansetron and local anesthetics, and finally contraindication for spinal anesthesia, lack of patient participation, sensitivity to ondansetron and local anesthetics, contraindication for spinal anesthesia, repeated<br>dural puncture, failed spinal anesthesia which requires adjuvants, and pre-pregnancy body mass index (BMI) less than 30.
**Shakhsemampour, 2018**	Dexamethasone<br>Placebo	17–46	Cesarean surgery; under spinal anesthesia.	Patients with prohibition of spinal anesthesia (for example, coagulation disorders, peripheral neuropathy, infection of the needle insertion site, and spinal cord disorders) and those with a history of headache, migraine, and hypertension (as in eclampsia and preeclampsia) were excluded.
**Shokrpour, 2018**	Ondansetron<br>Dexamethasone<br>Placebo	30.9±4.1<br>31.3±3.9<br>30.8±4.7	All pregnant women candidated for elective caesarean who did not agree to undergo spinal anesthesia； All pregnant women candidated for emergency caesarean. Patients who were abusive drug users.	All pregnant women candidated for elective caesarean whose spinal anesthesia had failed and were applying for general anesthesia; Mothers allergic to dexamethasone or ondansetron. All pregnant women candidated for elective caesarean under spinal anesthesia who had undergone spinal anesthesia more than twice.
**Dehghanpisheh, 2019**	Aminophylline<br>Ondansetron<br>Placebo	30.0±4.9	The pregnant women who were candidate for elective cesarean surgery under spinal anesthesia; whose anesthesia risk were classified as American Society of Anesthesiologists (ASA) Classes I and II (completely healthy or with a controlled disease)	Exclusion criteria included: pregnant women with prior history of any cardiovascular disease (including rhythm or rate disorder, etc.); migraine and pressure headaches; chronic or gestational hypertension; preeclampsia; seizure; liver or kidney failure; psychiatric problems; history of allergy to ondansetron, aminophylline or theophylline; history of drug addiction; more than one attempt of penetration for administering spinal anesthesia; failure of the spinal anesthesia and leading to general anesthesia.
**Yang, 2019**	Aminophylline<br>Placebo	27.2±4.6<br>29.2±5.5	ASA I or II, aged 24–38 years, G1P0 LOA<br>(i.e. G1, pregnant once, so the first pregnancy; P0, produced 0 times, so has not given birth previously; LOA, left occipital position of the fetus), undergoing elective caesarean section by combined spinal-epidural anesthesia.	(i) a history of intraspinal puncture; (ii) contraindications, such as coagulation dysfunction, spinal malformation and infection of puncture site; (iii) abnormal liver or kidney function; (iv) cardiovascular system diseases, respiratory system diseases and diseases of the nervous system; (v) a history of drug hypersensitivity; (vi) intraoperative blood loss > 500 ml; (vii) failure of the spinal canal puncture (epidural or spinal needle puncture failure)
**Anbarlouei, 2020**	Dexamethasone<br>Hydrocortisone<br>Placebo	27.7±5.7<br>26.3±5.8<br>26.6±5.3	ASA class I or II patients. Patients undergoing<br>elective cesarean section underwent spinal anesthesia.	The histories of coagulopathy, eclampsia, preeclampsia, seizures, and using special drugs were considered as exclusion criteria.
**Ogunsiji, 2020**	Hydrocortisone<br>Placebo	31.9±5.0<br>31.7±4.4	The age of 18 years and above and an ASA physical status I or II.	Exclusion criteria were unwillingness to consent, previous history of PDPH or history of chronic/recurrent headaches, history of allergy to hydrocortisone, peptic ulcer disease, diabetes mellitus, hypertensive disease of pregnancy or an active fungal, viral or bacterial infection.
**Karami, 2021**	Pregabalin<br>Placebo	28.5±5.8<br>27.2±5.7	Patient consent to participate in the study.	The history of migraine, patients with ASA III ASA IV, patients with a history of dural puncture more than<br>once, patients with an indication for emergency C-section, previous history of PDPH, contraindications of spinal anesthesia, block failure, or patients who need adjuvant injection due to incomplete block, patients with surgical complications such as atony and heavy bleeding or hysterectomy, patients who do not complete the 3-day follow-up period for any reason.
**Refky, 2021**	Propofol<br>Aminophylline<br>Placebo	25.2±4.4<br>25.0±4.5<br>27.4±4.7	Elective cesarean section under spinal anesthesia. Age; 21-35 years. Body mass index (BMI); less than 30 kg/m2. Physical status; American Society of Anesthesiologists<br>(ASA) physical status II	Parturient's refusal. Parturients subjected to emergency cesarean section. Parturients with history of migraine, chronic headache or previous PDPH. Parturients with history of analgesic consumption, substance abuse, and smoking. Parturients with chronic or gestational hypertension; preeclampsia and liver or kidney failure. Parturients with hypersensitivity to one of the used drugs. Parturients with cardiovascular diseases, respiratory system diseases and neurological and psychiatric disorders. More than one trial for administering spinal anesthesia or failure of spinal anesthesia. Parturients who suffered from massive blood loss. Parturients who suffered from intraoperative nausea and vomiting (IONV) immediately after spinal anesthesia or before delivery of the baby.
**Nikooseresht, 2022**	Magnesium<br>Placebo	30.8±8.6<br>30.5±6.0	Singleton healthy term parturients (with a gestational age of > 37 weeks) who were candidates for elective cesarean section and aged between 18 - 45 years.	Patients were excluded from the study if they used psychoactive drugs and had chronic hypertension, preeclampsia, history of migraine or chronic headache, and allergy to any of the study drugs. Failed spinal block and patients with more than one attempt for spinal anesthesia were also excluded from the study.
**Okpala, 2020**	Dexamethasone<br>Placebo	27.8±6.9<br>28.2±6.8	All parturients who underwent cesarean section under spinal anesthesia that were willing to participate in the trial gave an informed consent.	Women with history of dexamethasone intolerance or<br>past hypersensitivity to dexamethasone were excluded from the study. Those with past history of chronic headache or recent onset of acute headache were excluded. Those not willing to participate in the study were also excluded. Women who had multiple punctures (>2) during the spinal anesthesia procedure were excluded from the study.
**Razavizadeh, 2022**	Aminophylline<br>Dexamethasone<br>Placebo	27.4±5.1<br>27.6±4.7<br>28.3±4.7	Anesthesiologists (ASA) physical status I−II who underwent elective C-section with spinal anesthesia; pregnant women between the ages of 18 and 45 who underwent the elective C-section for the first time with spinal anesthesia	The exclusion criteria were history of previous headaches and migraines, presence of psychiatric problems, preeclampsia, coagulation disorders, peripheral neuropathy, spinal cord disorders, history of a prior surgery with spinal anesthesia, history of drug use and medication within 24 hours before C-section, history of peptic ulcer, arrhythmia, and seizures, converted spinal anesthesia to general anesthesia, history of allergy to aminophylline or dexamethasone, and a history of diabetes.

Table S3. Head-to-head comparisons of incidence of post-dural puncture headache at 48 hours after surgery

AMP							
0.44 (0.11, 1.35)	DXM						
4.14 (0.47, 35.12)	9.34 (1.23, 88.61)	GBP_PGB					
1.11 (0.15, 6.65)	2.51 (0.51, 13.7)	0.27 (0.02, 3.21)	HCT				
1.70 (0.19, 14.44)	3.82 (0.49, 36.61)	0.41 (0.03, 6.39)	1.52 (0.12, 20.85)	Mg			
0.93 (0.24, 3.39)	2.10 (0.54, 10.17)	0.22 (0.02, 2.17)	0.84 (0.12, 6.74)	0.55 (0.06, 5.48)	OND		
1.66 (0.2, 19.19)	3.84 (0.43, 53.71)	0.41 (0.02, 9.03)	1.53 (0.11, 29.65)	1.01 (0.06, 22.3)	1.81 (0.17, 25.83)	PPF	
0.51 (0.19, 1.23)	1.15 (0.52, 3.00)	0.12 (0.02, 0.82)	0.46 (0.09, 2.43)	0.30 (0.04, 2.08)	0.55 (0.16, 1.74)	0.30 (0.03, 2.42)	Placebo

AMP, aminophylline; COS, cosyntropin; DXM, dexamethasone; GBP/PGB, gabapentin or pregabalin; HCT, hydrocortisone; Mg, magnesium; OND, ondansetron; PPF, propofol.

Table S4. Head-to-head comparisons of incidence of post-dural puncture headache at 24 hours after surgery

AMP						
0.77 (0.15, 4.36)	DXM					
4.41 (0.16, 269.0)	5.62 (0.24, 289.1)	HCT				
1.89 (0.08, 44.69)	2.44 (0.10, 55.75)	0.42 (0.01, 31.19)	Mg			
0.73 (0.10, 5.27)	0.94 (0.10, 8.10)	0.16 (0.01, 6.00)	0.38 (0.01, 11.65)	OND		
0.89 (0.06, 13.07)	1.15 (0.06, 20.96)	0.20 (0.01, 12.44)	0.47 (0.01, 25.11)	1.22 (0.05, 29.81)	PPF	
0.48 (0.13, 1.67)	0.63 (0.16, 2.09)	0.11 (0.002, 2.42)	0.26 (0.01, 4.57)	0.67 (0.11, 4.06)	0.53 (0.04, 7.63)	Placebo

AMP, aminophylline; COS, cosyntropin; DXM, dexamethasone; HCT, hydrocortisone; Mg, magnesium; OND, ondansetron; PPF, propofol.

Table S5. Head-to-head comparisons of severity of post-dural puncture headache at 24 hours after surgery

AMP					
0.88 (-1.47, 4.15)	DXM				
1.75 (-1.90, 5.82)	0.81 (-2.68, 3.97)	GBP/PGB			
1.99 (-1.97, 6.31)	1.02 (-2.78, 4.55)	0.22 (-4.24, 4.70)	Mg		
2.83 (-0.01, 5.03)	1.85 (-1.03, 3.52)	1.01 (-2.96, 4.15)	0.76 (-3.46, 4.31)	OND	
0.97 (-1.27, 3.68)	0.09 (-1.63, 1.38)	-0.75 (-3.72, 2.2)	-0.97 (-4.34, 2.38)	-1.8 (-3.30, 0.60)	Placebo

AMP, aminophylline; DXM, dexamethasone; GBP/PGB, gabapentin or pregabalin; Mg, magnesium; OND, ondansetron; PPF, propofol. Data in red color means $P < 0.05$

Table S6. Head-to-head comparisons of severity of post-dural puncture headache at 48 hours after surgery

AMP					
1.43 (-1.63, 4.52)	DXM				
1.63 (-2.56, 5.90)	0.20 (-3.36, 3.78)	GBP/PGB			
1.88 (-2.79, 6.55)	0.45 (-3.66, 4.56)	0.25 (-4.72, 5.22)	Mg		
1.96 (-0.82, 4.71)	0.54 (-1.8, 2.81)	0.33 (-3.52, 4.09)	0.08 (-4.25, 4.37)	OND	
1.00 (-1.74, 3.78)	-0.43 (-1.98, 1.15)	-0.63 (-3.84, 2.58)	-0.88 (-4.66, 2.92)	-0.96 (-2.99, 1.14)	Placebo

AMP, aminophylline; COS, cosyntropin; DXM, dexamethasone; GBP/PGB, gabapentin or pregabalin; Mg, magnesium; OND, ondansetron.

Table S7. Head-to-head comparisons of severity of post-dural puncture headache at 72 hours after surgery

AMP							
-0.38 (-3.24, 2.46)	DXM						
1.14 (-2.24, 4.53)	1.52 (-2.15, 5.20)	GBP/PGB					
-0.33 (-4.56, 3.91)	0.05 (-4.43, 4.54)	-1.47 (-6.08, 3.15)	HCT				
-0.02 (-4.87, 4.86)	0.37 (-4.71, 5.44)	-1.16 (-6.33, 4.02)	0.32 (-5.48, 6.09)	Mg			
1.76 (-1.20, 4.56)	2.14 (-1.43, 5.55)	0.62 (-3.20, 4.31)	2.09 (-2.53, 6.57)	1.78 (-3.41, 6.82)	OND		
1.82 (-1.55, 5.16)	2.20 (-1.84, 6.23)	0.67 (-3.64, 4.98)	2.14 (-2.88, 7.15)	1.83 (-3.72, 7.36)	0.06 (-3.96, 4.17)	PPF	
-0.36 (-2.40, 1.69)	0.02 (-2.47, 2.54)	-1.50 (-4.19, 1.19)	-0.03 (-3.75, 3.71)	-0.35 (-4.76, 4.06)	-2.13 (-4.63, 0.55)	-2.18 (-5.51, 1.18)	Placebo

AMP, aminophylline; DXM, dexamethasone; GBP/PGB, gabapentin or pregabalin; HCT, hydrocortisone; Mg, magnesium; OND, ondansetron; PPF, propofol.

Table S8. Head-to-head comparisons of incidence of postoperative nausea and vomiting

AMP						
0.71 (0.1, 3.66)	DXM					
0.91 (0.15, 4.87)	1.28 (0.27, 7.42)	GBP/PGB				
0.35 (0.03, 3.54)	0.49 (0.06, 5.46)	0.39 (0.04, 3.71)	HCT			
5.5 (0.61, 44.35)	7.79 (1.07, 67.48)	6.03 (0.8, 45.7)	15.67 (1.18, 194.95)	OND		
9.04 (1.67, 53.56)	12.9 (2.17, 106.45)	9.99 (1.66, 72.17)	25.94 (2.36, 312.55)	1.65 (0.19, 17.04)	PPF	
0.64 (0.17, 2.34)	0.90 (0.30, 3.47)	0.70 (0.24, 2.23)	1.81 (0.26, 12.57)	0.12 (0.02, 0.63)	0.07 (0.01, 0.30)	Placebo

AMP, aminophylline; DXM, dexamethasone; GBP/PGB, gabapentin or pregabalin; HCT, hydrocortisone; OND, ondansetron; PPF, propofol. Data in red color means $P < 0.05$

Table S9. Assessment of publication bias for network meta-analysis

Outcome	Test of Publication Bias Begg's *P* value	Test of Publication Bias Egger's *P* value
Cumulative incidence of post-dural puncture headache during the follow-up period	0.318	0.182
Incidence of post-dural puncture headache at 24 hours after surgery	0.278	0.128
Incidence of post-dural puncture headache at 48 hours after surgery	0.721	0.823
Severity of post-dural puncture headache at 24 hours after surgery	0.755	0.442
Severity of post-dural puncture headache at 48 hours after surgery	0.585	0.455
Severity of post-dural puncture headache at 72 hours after surgery	0.804	0.226
Postoperative nausea and vomiting	0.827	0.995

Figure S1. Risk of bias summary

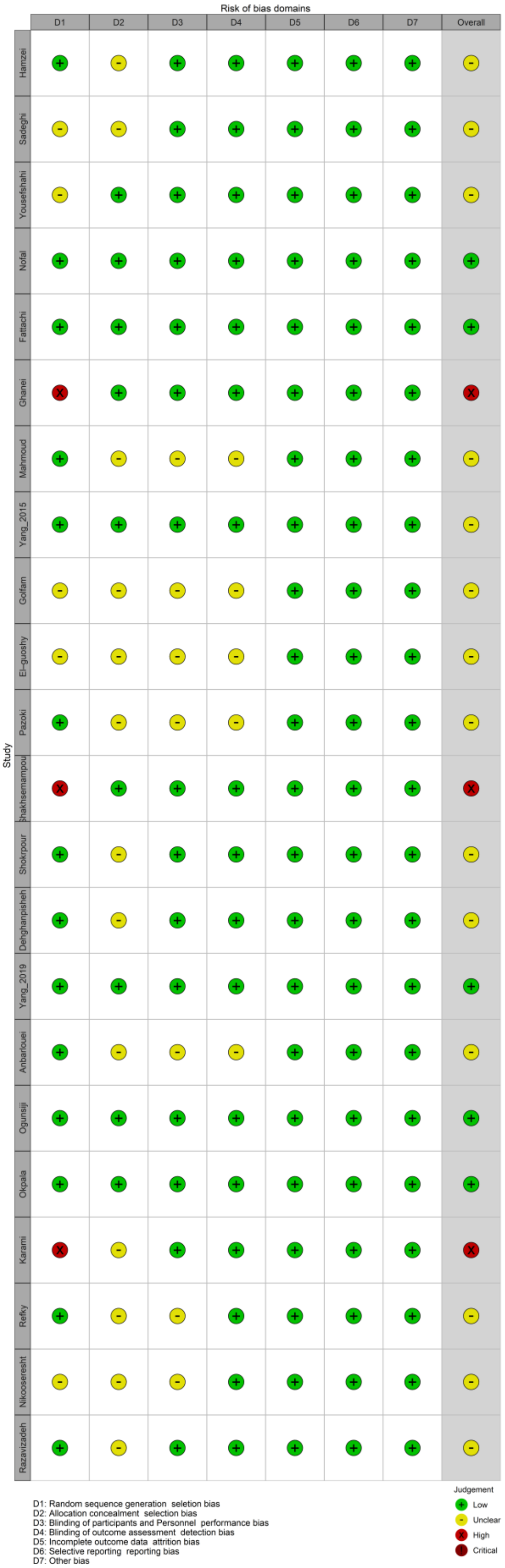

Figure S2. Risk of bias graph

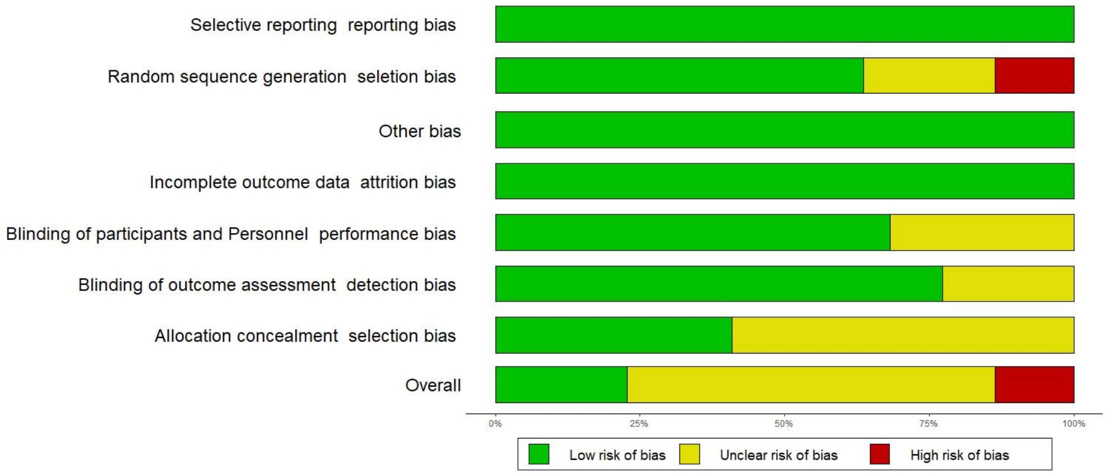

Figure S3. Inconsistency test of cumulative incidence of post-dural puncture headache within 7 days

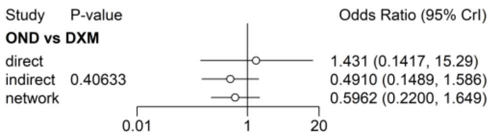

Figure S4. Inconsistency test of incidence of post-dural puncture headache at 24 hours after surgery

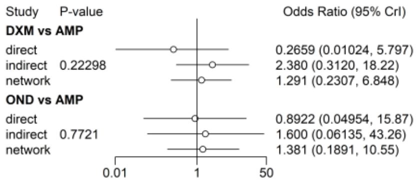

Figure S5. Inconsistency test of incidence of post-dural puncture headache at 48 hours after surgery

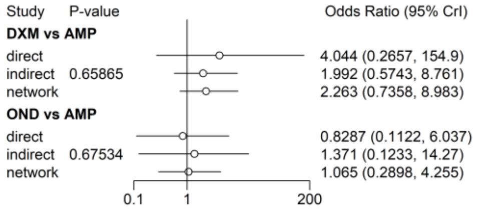

Figure S6. Inconsistency test of severity of post-dural puncture headache at 24 hours after surgery

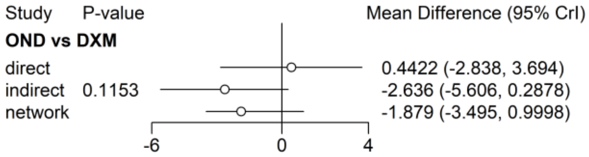

Figure S7. Inconsistency test of severity of post-dural puncture headache at 48 hours after surgery

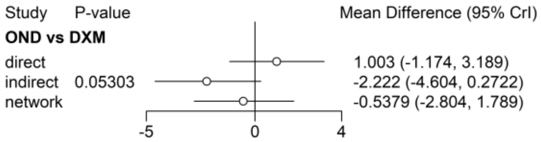

Figure S8. Inconsistency test of severity of post-dural puncture headache at 72 hours after surgery

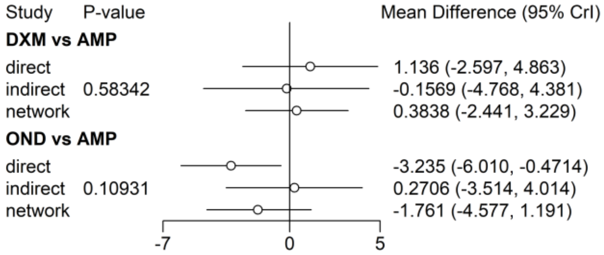

Figure S9. Inconsistency test of postoperative nausea and vomiting

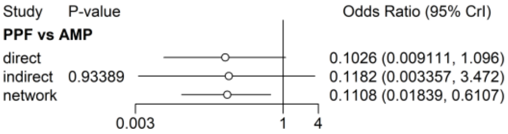

Figure S10. Funnel plot of the outcomes

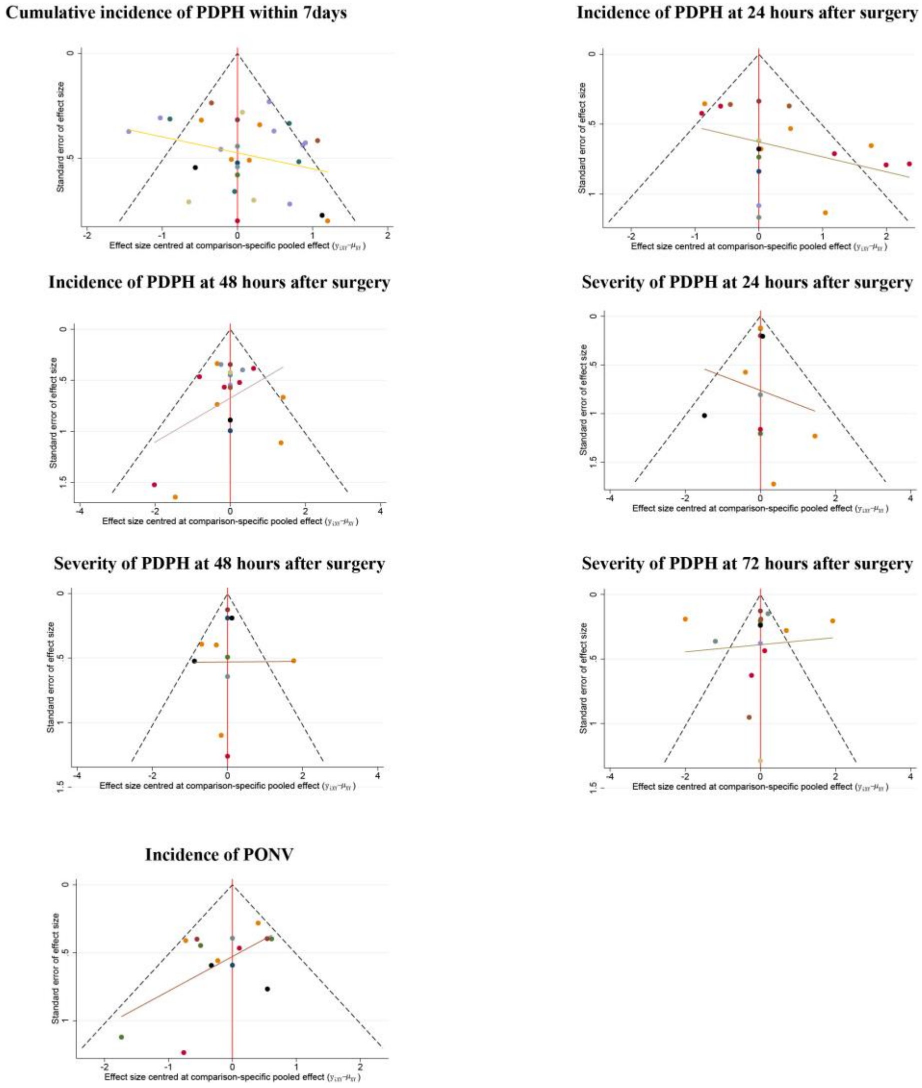

Supplement: Supplementary file 2 — Additional file 2: Table S1. Strategy of this meta-analysis. Table S2. Inclusion and exclusion criteria in each involved study. Table S3. Head-to-head comparisons of incidence of post-dural puncture headache at 48 hours after surgery. Table S4. Head-to-head comparisons of incidence of post-dural puncture headache at 24 hours after surgery. Table S5. Head-to-head comparisons of severity of post-dural puncture headache at 24 hours after surgery. Table S6. Head-to-head comparisons of severity of post-dural puncture headache at 48 hours after surgery. Table S7. Head-to-head comparisons of severity of post-dural puncture headache at 72 hours after surgery. Table S8. Head-to-head comparisons of incidence of postoperative nausea and vomiting. Table S9. Assessment of publication bias for network meta-analysis. Figure S1. Risk of bias summary. Figure S2. Risk of bias graph. Figure S3. Inconsistency test of cumulative incidence of post-dural puncture headache within 7 days. Figure S4. Inconsistency test of incidence of post-dural puncture headache at 24 hours after surgery. Figure S5. Inconsistency test of incidence of post-dural puncture headache at 48 hours after surgery. Figure S6. Inconsistency test of severity of post-dural puncture headache at 24 hours after surgery. Figure S7. Inconsistency test of severity of post-dural puncture headache at 48 hours after surgery. Figure S8. Inconsistency test of severity of post-dural puncture headache at 72 hours after surgery. Figure S9. Inconsistency test of postoperative nausea and vomiting. Figure S10. Funnel plot of the outcomes. [file 12884_2023_5531_MOESM2_ESM.pdf]
